# Materials for Finishing and Polishing Fillings

**Published:** 1885-12

**Authors:** C. F. W. Bodecker


### MATERIALS FOR FINISHING AND POLISHING FILLINGS.

BY C. F. W. BODECKER, D. D. S., M. D. S.

The best filling, unless great care has been exercised in finishing, may result in a failure, especially when situated upon the proximate surface of a tooth. With most of the appliances in use to-day, to obtain the desired result involves a great deal of patient labor. In dressing down gold fillings, small shellac corundum points are very serviceable, but, as soon as an instrument made of this material is small enough to reach all the depressions upon the teeth, it readily wears out or breaks. For the proximate surfaces, sand, emery, corundum, etc., fastened upon paper, cloth, or celluloid, cut into strips or disks, have been employed, but with some difficulty, as the polishing materials are held upon the paper, etc., mostly by glue, and the cutting surface is destroyed as soon as it comes in contact with the saliva. Although an application of an alcoholic solution of shellac to the sand or emery paper will materially improve its durability, yet it remains a very disagreeable and sticky material to handle in the mouth, unless it can be kept perfectly dry.

Besides these, there are several other appliances, viz., Brown's polishing silver strips, copper strips, wood shavings, etc., but neither is free from objections. Not satisfied with these materials, and desirous of obtaining something better, Dr. Wm. Herbst, of Bremen, Dr. Franz Berggren, and Dr. E. Förberg, of Stockholm, have largely experimented in this direction, and in certain appliances have made considerable improvements, which deserve the acknowledgment of the profession.

It is well known that points and disks, made of black rubber and corundum, or emery, have been used many years, but they cannot be obtained from the dental depots (as is understood), on account of a pending patent law-suit. But every dental practitioner may easily prepare them for himself, and better ones than can be bought at present. For this purpose take one part of ordinary red rubber, and two parts of corundum, or emery. Warm the rubber upon a water bath, and gradually knead the corundum or emery into it, so that it is evenly distributed throughout the rubber, which is then flattened out so that it may be readily cut. Take button moulds, or points made of wood, of the required size and shape, fasten them with wax upon the ends of worn out engine burs, insert them into an ordinary deep rubber flask, head upwards, in such a manner that the upper surfaces of the points are exposed, and pour the counterpart. When the plaster has set, open the flask, remove the points or button moulds from the mandrels, and after they have been cleaned perfectly with boiling water, pack a little ordinary red rubber in the center around the mandrel, and fill the rest with the rubber impregnated with corundum or emery. Then close the flask, and vulcanize in the usual way. When the points are vulcanized they are, while rotating in the engine, shaped upon a coarse file, and then immersed in nitric acid from two to six hours, according to their thickness. But precaution must be taken to apply a thin coating of wax all over the mandrel, else the acid will dissolve the steel. When hard rubber, corundum, or emery points are treated in this manner, the nitric acid dissolves the outer layer of the rubber, leaving the corundum or emery intact, thus exposing a cutting surface superior to the best ordinary corundum point, or sharp steel bur, and a great deal more durable than either. (Dr. Wm. Herbst.)

Soft points and disks may be very easily made of ordinary velum rubber impregnated with powdered pumice, but as the soft rubber cannot well be fastened upon a mandrel, a hard center may be put into it in the following way: With a punch cut the disk out of a sheet of velum (soft) rubber, and out of this disk remove the center by means of a smaller punch. The center is replaced by a piece cut out of a sheet of unvulcanized hard rubber. The disks or points are then closely wrapped in tin foil, put in the flask, vulcanized, mounted, and trimmed upon coarse sandpaper, while rotating in the engine. (Dr. Berggren.)

To prepare disks that will withstand moisture, take a piece of strong, thin linen, and a piece of sand or emery paper. Varnish both with a rather thick solution of shellac in alcohol (the paper upon its sanded side), bring both together, and keep them under a press for three days. Then immerse in water, when the paper will be found to separate from the linen, leaving the sand or emery held by the shellac upon the linen, out of which the disks are stamped. (Dr. Wm. Herbst.)

Durable paper disks, with emery or corundum, may be prepared by coating thin cardboard (without gloss), as postal cards, with a thick solution of shellac, and sprinkling the thinnest possible layer of corundum or emery upon it, out of which, when perfectly dry, disks may be cut. (Dr. Förberg.)

To make disks of thin rubber cloth, to be used with advantage for polishing with powdered pumice or chalk, take two pieces of rubber cloth, as obtained in the rubber stores, coat them with a rather thick solution of shellac in alcohol, and immediately bring them together and keep them under a press for about two days. When thoroughly dry the sheet may be cut into disks by means of a punch, and when mounted upon a screw mandrel they can be made quite thin by holding them, while rotating in the engine, upon a piece of sandpaper. A variety of thicknesses of rubber cloth may be employed with advantage, but it is better to cement two layers together, and then only use one thickness of rubber cloth, as the shellac imparts great stiffness to the disk. (Dr. Wm. Herbst.)

For removing surplus filling materials from the proximal surfaces of teeth, a watch spring, upon which a layer of corundum or emery has been attached, is of great service. To prepare this, warm a thin watch spring over a Bunsen burner, or the flame of a spirit lamp, apply a thin coating of solid shellac, and quickly, while the shellac is yet in a fluid condition, immerse the spring into powdered corundum or emery. When perfectly cold they may be used in a saw frame, and will be found more serviceable than thin files. (Dr. Berggren.)

For polishing proximate surfaces of the teeth, ordinary rubber cloth cut into strips, or very narrow velvet braid, will be found to work admirably. Thin chamois leather will probably produce the finest polish, but when narrow strips are used they will stretch out, and tear very quickly. To overcome this difficulty, sew a seam lengthwise in the strip with a sewing machine, which will very materially strengthen it. (Dr. Berggren.)

To prepare tape which is very serviceable, and which will retain the polishing materials well, take some strong and thin linen tape, of desired breadth, soak in ordinary thin rubber cement for two or three days, then remove it, at the same time scraping off all the surplus rubber cement, and let it dry for about twenty-four hours. The tape is then impregnated by rubbing it with powdered corundum, pumice, chalk or any material that it is desired to use. The rubber cement will hold these substances very firmly in the meshes of the tape, and it may be used under water or saliva without losing its cutting surface. (Dr. Wm. Herbst.)

